# Supplementary material for: A Likelihood Approach for Real-Time Calibration of Stochastic Compartmental Epidemic Models
Source: PLoS Comput Biol. 2017 Jan 17;13(1):e1005257. doi: 10.1371/journal.pcbi.1005257 (PMC5240920; doi:10.1371/journal.pcbi.1005257)
Supplement: S1 File — (TAR.GZ) [file pcbi.1005257.s014.tar.gz › HSPH_Online-SI-Revision/PF08/n1-mild/n1-mild_PF2_table0.pdf]

1	newly 3 weeks spec	14.	29.74	{9., 55.}	128.	0.388401	newly 3 weeks cumul	75.	99.24	{37., 189.}	60.64	0.234999	newly 5 weeks spec	14.	21.61	{5., 40.}	86.2143	0.336483	newly 5 weeks cumul	112.	146.36	{59., 275.}	60.375	0.241584	newly 55 weeks cumul	214.	269.72	{80., 459.}	61.6355	0.278032
2	newly 3 weeks spec	8.	8.64	{0., 21.}	94.75	0.695254	newly 3 weeks cumul	39.	28.72	{0., 73.}	69.8974	0.680206	newly 5 weeks spec	6.	6.01	{0., 15.}	100.833	0.775202	newly 5 weeks cumul	52.	41.59	{0., 107.}	74.5962	0.710457	newly 51 weeks cumul	74.	82.43	{0., 189.}	100.473	0.785341
3	newly 3 weeks spec	19.	29.02	{1., 66.}	98.9474	0.488842	newly 3 weeks cumul	66.	97.02	{5., 192.}	91.5758	0.425095	newly 5 weeks spec	13.	23.2	{0., 56.}	128.462	0.5627	newly 5 weeks cumul	88.	146.91	{8., 285.}	107.148	0.440938	newly 60 weeks cumul	152.	288.57	{22., 586.}	131.27	0.496188
4	newly 3 weeks spec	7.	22.84	{6., 46.}	238.286	0.496915	newly 3 weeks cumul	27.	76.62	{23., 160.}	192.296	0.41658	newly 5 weeks spec	12.	18.17	{2., 38.}	93.4167	0.411301	newly 5 weeks cumul	48.	115.46	{31., 235.}	154.458	0.375871	newly 54 weeks cumul	336.	222.79	{35., 432.}	47.3423	0.374443
5	newly 3 weeks spec	21.	21.08	{2., 52.}	69.7143	0.460373	newly 3 weeks cumul	87.	72.05	{7., 164.}	59.1379	0.423862	newly 5 weeks spec	13.	15.81	{0., 39.}	92.8462	0.557522	newly 5 weeks cumul	118.	106.02	{13., 237.}	62.3051	0.42994	newly 56 weeks cumul	175.	204.05	{18., 488.}	81.0571	0.465172
6	newly 3 weeks spec	25.	31.19	{1., 66.}	84.52	0.532046	newly 3 weeks cumul	80.	100.83	{5., 201.}	82.8375	0.457438	newly 5 weeks spec	28.	25.99	{0., 57.}	62.75	0.541999	newly 5 weeks cumul	143.	155.34	{10., 319.}	70.028	0.443819	newly 60 weeks cumul	316.	324.91	{35., 593.}	54.8639	0.375129
7	newly 3 weeks spec	43.	26.25	{2., 60.}	56.6744	0.520442	newly 3 weeks cumul	128.	84.94	{9., 167.}	51.7656	0.427284	newly 5 weeks spec	19.	19.91	{0., 43.}	73.4211	0.536014	newly 5 weeks cumul	180.	128.43	{11., 261.}	52.8944	0.415587	newly 59 weeks cumul	287.	261.92	{33., 541.}	57.8467	0.400859
8	newly 3 weeks spec	17.	14.59	{0., 33.}	64.2941	0.553506	newly 3 weeks cumul	45.	51.07	{5., 105.}	68.6444	0.370156	newly 5 weeks spec	19.	11.83	{0., 26.}	59.2105	0.660677	newly 5 weeks cumul	80.	76.56	{5., 159.}	61.6	0.411119	newly 51 weeks cumul	385.	155.06	{7., 359.}	60.6286	0.690882
9	newly 3 weeks spec	39.	14.23	{2., 26.}	65.1026	0.698491	newly 3 weeks cumul	109.	47.85	{7., 81.}	58.7798	0.539551	newly 5 weeks spec	28.	11.46	{0., 23.}	62.5714	0.74364	newly 5 weeks cumul	160.	71.48	{10., 125.}	58.9375	0.556177	newly 50 weeks cumul	206.	143.11	{12., 302.}	57.5485	0.521929
10	newly 3 weeks spec	14.	16.64	{2., 29.}	68.1429	0.379435	newly 3 weeks cumul	60.	58.15	{10., 103.}	49.55	0.300615	newly 5 weeks spec	11.	12.73	{0., 28.}	77.3636	0.509234	newly 5 weeks cumul	94.	85.76	{13., 147.}	50.234	0.333121	newly 51 weeks cumul	109.	169.93	{15., 343.}	103.312	0.435936
11	newly 3 weeks spec	16.	11.67	{0., 25.}	62.5625	0.648624	newly 3 weeks cumul	57.	40.47	{2., 88.}	58.2632	0.527031	newly 5 weeks spec	6.	8.78	{0., 24.}	118.667	0.683735	newly 5 weeks cumul	78.	59.88	{3., 134.}	60.9744	0.549571	newly 50 weeks cumul	387.	117.31	{3., 292.}	69.9871	0.925748
12	newly 3 weeks spec	18.	13.48	{0., 29.}	60.	0.572643	newly 3 weeks cumul	54.	45.47	{5., 93.}	57.5	0.438798	newly 5 weeks spec	20.	9.86	{0., 24.}	62.1	0.78519	newly 5 weeks cumul	85.	67.48	{6., 139.}	58.4235	0.479993	newly 51 weeks cumul	271.	136.35	{6., 340.}	60.7491	0.687781
13	newly 3 weeks spec	19.	11.08	{0., 23.}	63.3684	0.706847	newly 3 weeks cumul	55.	38.82	{1., 89.}	61.5273	0.570773	newly 5 weeks spec	8.	7.37	{0., 14.}	84.875	0.747	newly 5 weeks cumul	79.	54.75	{1., 125.}	64.1646	0.617063	newly 52 weeks cumul	97.	97.47	{1., 197.}	83.	0.641996
14	newly 3 weeks spec	15.	18.75	{2., 43.}	84.8667	0.480795	newly 3 weeks cumul	78.	63.	{8., 135.}	58.7436	0.435527	newly 5 weeks spec	10.	14.48	{0., 36.}	110.6	0.567131	newly 5 weeks cumul	112.	93.68	{10., 206.}	61.4286	0.455557	newly 56 weeks cumul	131.	182.2	{12., 424.}	98.2748	0.485745
15	newly 3 weeks spec	27.	30.73	{15., 49.}	43.2222	0.166137	newly 3 weeks cumul	78.	98.1	{45., 156.}	46.5897	0.166447	newly 5 weeks spec	19.	24.77	{8., 42.}	58.8947	0.223673	newly 5 weeks cumul	124.	151.1	{73., 245.}	44.1774	0.162016	newly 56 weeks cumul	280.	327.37	{128., 559.}	48.6107	0.198579
16	newly 3 weeks spec	11.	22.07	{5., 40.}	127.364	0.418798	newly 3 weeks cumul	35.	74.59	{24., 133.}	128.314	0.353978	newly 5 weeks spec	16.	17.23	{2., 33.}	62.8125	0.406367	newly 5 weeks cumul	59.	111.09	{29., 208.}	109.339	0.340006	newly 54 weeks cumul	273.	220.52	{29., 406.}	50.9597	0.375593
17	newly 3 weeks spec	20.	32.35	{11., 62.}	83.05	0.25778	newly 3 weeks cumul	50.	103.85	{46., 183.}	113.26	0.292952	newly 5 weeks spec	12.	26.44	{7., 49.}	137.	0.37241	newly 5 weeks cumul	78.	160.6	{66., 281.}	114.231	0.300457	newly 57 weeks cumul	269.	347.67	{100., 568.}	59.9665	0.246131
18	newly 3 weeks spec	14.	13.49	{1., 28.}	63.3571	0.450704	newly 3 weeks cumul	53.	47.21	{8., 93.}	55.4906	0.369863	newly 5 weeks spec	18.	9.27	{0., 21.}	61.2778	0.682336	newly 5 weeks cumul	81.	67.81	{9., 138.}	55.4938	0.40295	newly 50 weeks cumul	239.	128.35	{9., 297.}	57.7782	0.593134
19	newly 3 weeks spec	24.	24.18	{1., 53.}	67.8333	0.517178	newly 3 weeks cumul	86.	81.04	{8., 164.}	58.3721	0.409337	newly 5 weeks spec	24.	17.33	{1., 43.}	61.625	0.586086	newly 5 weeks cumul	128.	118.39	{9., 239.}	58.3359	0.419326	newly 57 weeks cumul	218.	218.31	{13., 513.}	63.0138	0.441653
20	newly 3 weeks spec	15.	22.68	{7., 47.}	76.6667	0.259916	newly 3 weeks cumul	66.	75.95	{31., 145.}	49.5303	0.204013	newly 5 weeks spec	18.	17.36	{5., 33.}	53.6667	0.301247	newly 5 weeks cumul	102.	113.68	{37., 196.}	48.6863	0.20659	newly 54 weeks cumul	153.	227.35	{59., 452.}	81.8627	0.292984
21	newly 3 weeks spec	19.	13.58	{0., 30.}	71.6842	0.703416	newly 3 weeks cumul	59.	45.78	{3., 101.}	65.0169	0.545847	newly 5 weeks spec	16.	9.51	{0., 26.}	76.5625	0.893325	newly 5 weeks cumul	93.	66.47	{3., 140.}	66.6129	0.606169	newly 54 weeks cumul	145.	120.21	{3., 322.}	78.7379	0.681584
22	newly 3 weeks spec	17.	28.13	{5., 61.}	100.529	0.37926	newly 3 weeks cumul	45.	94.73	{30., 202.}	127.533	0.332166	newly 5 weeks spec	12.	22.47	{1., 53.}	127.083	0.512048	newly 5 weeks cumul	68.	142.44	{32., 300.}	130.588	0.353717	newly 56 weeks cumul	388.	289.89	{33., 566.}	48.7964	0.379194
23	newly 3 weeks spec	18.	15.48	{3., 30.}	49.7778	0.38556	newly 3 weeks cumul	69.	54.07	{10., 95.}	41.6377	0.304097	newly 5 weeks spec	27.	12.16	{1., 24.}	59.4815	0.618963	newly 5 weeks cumul	118.	80.02	{19., 142.}	44.8136	0.354809	newly 51 weeks cumul	241.	160.87	{20., 328.}	52.0124	0.451431
24	newly 3 weeks spec	25.	18.51	{2., 39.}	52.92	0.430404	newly 3 weeks cumul	83.	63.52	{13., 127.}	48.8675	0.325241	newly 5 weeks spec	20.	14.4	{0., 32.}	57.9	0.581816	newly 5 weeks cumul	121.	94.9	{14., 180.}	49.0744	0.336463	newly 53 weeks cumul	298.	181.44	{22., 370.}	52.4765	0.457089
25	newly 3 weeks spec	12.	12.78	{0., 30.}	71.6667	0.500833	newly 3 weeks cumul	34.	43.61	{4., 91.}	76.3235	0.384491	newly 5 weeks spec	11.	9.56	{0., 25.}	72.3636	0.588964	newly 5 weeks cumul	59.	63.77	{5., 136.}	66.5254	0.411467	newly 50 weeks cumul	239.	129.04	{5., 289.}	59.3473	0.634196
26	newly 3 weeks spec	22.	23.14	{4., 46.}	56.1818	0.314249	newly 3 weeks cumul	61.	77.18	{24., 141.}	59.7705	0.238381	newly 5 weeks spec	13.	17.95	{3., 38.}	87.6154	0.393225	newly 5 weeks cumul	91.	116.57	{31., 225.}	64.5165	0.259035	newly 53 weeks cumul	122.	226.12	{40., 431.}	114.295	0.347696
27	newly 3 weeks spec	11.	12.97	{0., 29.}	78.4545	0.531363	newly 3 weeks cumul	44.	45.57	{2., 92.}	63.1591	0.455267	newly 5 weeks spec	10.	10.03	{0., 23.}	78.1	0.641668	newly 5 weeks cumul	72.	67.89	{2., 137.}	61.0417	0.50044	newly 50 weeks cumul	169.	134.55	{2., 289.}	60.8225	0.613192
28	newly 3 weeks spec	15.	27.13	{1., 58.}	117.133	0.500348	newly 3 weeks cumul	48.	87.68	{17., 178.}	105.5	0.335284	newly 5 weeks spec	16.	22.52	{0., 46.}	88.	0.496906	newly 5 weeks cumul	77.	135.04	{18., 284.}	103.221	0.35365	newly 56 weeks cumul	293.	269.94	{18., 516.}	55.0785	0.386942
29	newly 3 weeks spec	19.	21.19	{5., 37.}	58.0526	0.306494	newly 3 weeks cumul	60.	70.62	{25., 115.}	51.6667	0.238752	newly 5 weeks spec	14.	16.65	{3., 29.}	67.5	0.359013	newly 5 weeks cumul	96.	105.75	{37., 172.}	49.8646	0.251563	newly 53 weeks cumul	143.	205.17	{45., 403.}	81.2098	0.33199
30	newly 3 weeks spec	28.	22.79	{2., 52.}	60.4643	0.462126	newly 3 weeks cumul	82.	72.53	{9., 152.}	57.4024	0.374003	newly 5 weeks spec	11.	16.98	{1., 43.}	104.727	0.457032	newly 5 weeks cumul	114.	108.71	{14., 233.}	60.2193	0.369305	newly 57 weeks cumul	214.	223.82	{29., 519.}	69.8131	0.409602
31	newly 3 weeks spec	34.	18.75	{1., 42.}	58.5	0.570991	newly 3 weeks cumul	96.	66.77	{9., 146.}	55.1979	0.422664	newly 5 weeks spec	13.	13.9	{1., 30.}	71.3846	0.473943	newly 5 weeks cumul	127.	96.57	{15., 214.}	55.5984	0.413481	newly 55 weeks cumul	143.	166.5	{18., 398.}	74.9231	0.404586
32	newly 3 weeks spec	11.	20.37	{0., 41.}	120.455	0.505547	newly 3 weeks cumul	53.	67.42	{9., 130.}	68.7925	0.384738	newly 5 weeks spec	12.	16.77	{0., 37.}	91.25	0.541157	newly 5 weeks cumul	70.	102.77	{9., 199.}	84.8714	0.421038	newly 53 weeks cumul	339.	211.09	{10., 417.}	49.6726	0.541221
33	newly 3 weeks spec	38.	24.36	{7., 47.}	46.5263	0.376461	newly 3 weeks cumul	103.	83.18	{29., 147.}	41.8641	0.264593	newly 5 weeks spec	18.	19.52	{2., 38.}	61.8889	0.402271	newly 5 weeks cumul	138.	124.89	{40., 236.}	43.8913	0.263649	newly 54 weeks cumul	205.	236.4	{44., 441.}	60.5756	0.312683
34	newly 3 weeks spec	13.	19.57	{6., 35.}	74.6923	0.280462	newly 3 weeks cumul	28.	64.6	{27., 103.}	141.214	0.369128	newly 5 weeks spec	11.	15.24	{3., 29.}	72.3636	0.345767	newly 5 weeks cumul	49.	96.84	{35., 163.}	111.51	0.326046	newly 53 weeks cumul	176.	202.15	{43., 383.}	56.7443	0.281707
35	newly 3 weeks spec	36.	27.4	{9., 54.}	46.6111	0.321586	newly 3 weeks cumul	89.	90.98	{38., 176.}	47.1461	0.229656	newly 5 weeks spec	34.	21.39	{5., 43.}	48.9118	0.389024	newly 5 weeks cumul	162.	136.02	{49., 265.}	43.1111	0.25409	newly 55 weeks cumul	281.	267.22	{68., 484.}	43.1174	0.257725
36	newly 3 weeks spec	29.	24.94	{1., 56.}	60.4138	0.493292	newly 3 weeks cumul	101.	82.06	{8., 177.}	55.8218	0.40673	newly 5 weeks spec	18.	18.88	{1., 45.}	72.4444	0.494547	newly 5 weeks cumul	133.	122.2	{12., 271.}	59.3835	0.394005	newly 57 weeks cumul	143.	225.45	{19., 514.}	99.	0.408253
37	newly 3 weeks spec	16.	24.34	{7., 45.}	81.125	0.314857	newly 3 weeks cumul	67.	80.98	{25., 136.}	56.2687	0.244452	newly 5 weeks spec	19.	19.55	{3., 39.}	56.2632	0.366238	newly 5 weeks cumul	103.	122.49	{33., 226.}	56.3592	0.257438	newly 53 weeks cumul	179.	252.28	{47., 467.}	76.8939	0.324718
38	newly 3 weeks spec	31.	22.8	{0., 56.}	63.7419	0.614493	newly 3 weeks cumul	127.	74.86	{4., 173.}	56.5512	0.583481	newly 5 weeks spec	22.	16.41	{0., 42.}	66.9545	0.624407	newly 5 weeks cumul	168.	110.39	{8., 254.}	58.1726	0.567478	newly 57 weeks cumul	211.	203.85	{8., 460.}	68.0237	0.541043
39	newly 3 weeks spec	16.	16.13	{0., 38.}	73.5625	0.54046	newly 3 weeks cumul	52.	57.78	{7., 128.}	72.2308	0.406364	newly 5 weeks spec	14.	13.	{0., 30.}	71.1429	0.590394	newly 5 weeks cumul	79.	85.37	{8., 182.}	72.1139	0.431547	newly 55 weeks cumul	143.	170.05	{8., 391.}	85.6853	0.503983
40	newly 3 weeks spec	28.	24.68	{1., 57.}	62.2857	0.535105	newly 3 weeks cumul	115.	81.97	{5., 175.}	56.7913	0.512307	newly 5 weeks spec	12.	20.11	{0., 44.}	123.917	0.591442	newly 5 weeks cumul	156.	123.57	{8., 272.}	59.2628	0.504786	newly 58 weeks cumul	187.	242.31	{9., 523.}	87.9947	0.513013
41	newly 3 weeks spec	19.	29.38	{2., 60.}	100.947	0.499865	newly 3 weeks cumul	73.	94.87	{5., 201.}	80.0959	0.436992	newly 5 weeks spec	10.	23.25	{0., 50.}	174.3	0.62649	newly 5 weeks cumul	107.	144.66	{8., 301.}	84.2991	0.449536	newly 59 weeks cumul	145.	296.22	{11., 598.}	142.138	0.530634
42	newly 3 weeks spec	12.	29.05	{1., 64.}	173.083	0.548034	newly 3 weeks cumul	104.	98.09	{9., 223.}	56.5673	0.395311	newly 5 weeks spec	0.1	21.73	{0., 48.}	21650.	1.98526	newly 5 weeks cumul	106.	144.28	{12., 320.}	80.3396	0.399488	newly 58 weeks cumul	106.	255.05	{20., 529.}	172.104	0.520906
43	newly 3 weeks spec	28.	35.63	{6., 64.}	67.6071	0.38827	newly 3 weeks cumul	117.	123.05	{27., 203.}	48.6068	0.314056	newly 5 weeks spec	11.	27.3	{2., 56.}	179.818	0.536064	newly 5 weeks cumul	151.	180.93	{35., 308.}	58.5894	0.323944	newly 60 weeks cumul	167.	304.97	{58., 624.}	109.419	0.399164
44	newly 3 weeks spec	20.	17.93	{3., 34.}	48.55	0.340481	newly 3 weeks cumul	78.	60.04	{21., 99.}	41.4872	0.295919	newly 5 weeks spec	6.	12.8	{2., 25.}	144.	0.447223	newly 5 weeks cumul	92.	87.66	{29., 152.}	43.6957	0.276505	newly 52 weeks cumul	103.	170.13	{41., 338.}	98.3398	0.376588
45	newly 3 weeks spec	25.	17.5	{5., 35.}	50.48	0.384765	newly 3 weeks cumul	80.	59.74	{25., 102.}	44.05	0.273808	newly 5 weeks spec	16.	13.84	{3., 27.}	49.	0.340536	newly 5 weeks cumul	114.	89.06	{35., 148.}	43.7368	0.273026	newly 50 weeks cumul	184.	184.66	{45., 372.}	54.2174	0.304134
46	newly 3 weeks spec	21.	34.4	{14., 59.}	79.9048	0.261824	newly 3 weeks cumul	65.	110.45	{50., 181.}	80.0769	0.238604	newly 5 weeks spec	19.	27.03	{9., 51.}	64.2632	0.237653	newly 5 weeks cumul	90.	168.1	{79., 276.}	94.0222	0.263989	newly 57 weeks cumul	370.	336.8	{126., 542.}	34.827	0.192299
47	newly 3 weeks spec	10.	23.41	{5., 46.}	152.7	0.407049	newly 3 weeks cumul	30.	76.4	{22., 129.}	166.2	0.395734	newly 5 weeks spec	5.	18.78	{2., 35.}	298.8	0.618515	newly 5 weeks cumul	45.	116.45	{41., 221.}	171.267	0.409466	newly 55 weeks cumul	300.	239.7	{50., 441.}	47.6067	0.335002
48	newly 3 weeks spec	18.	15.2	{1., 28.}	52.2222	0.425826	newly 3 weeks cumul	78.	49.75	{13., 88.}	46.6282	0.363469	newly 5 weeks spec	4.	12.98	{0., 28.}	258.5	0.633814	newly 5 weeks cumul	93.	76.28	{18., 135.}	47.1398	0.335257	newly 50 weeks cumul	172.	158.56	{19., 323.}	62.1279	0.426561
49	newly 3 weeks spec	6.	21.89	{3., 46.}	282.167	0.595257	newly 3 weeks cumul	35.	71.16	{22., 137.}	118.914	0.335061	newly 5 weeks spec	12.	17.41	{1., 34.}	91.0833	0.467811	newly 5 weeks cumul	58.	108.43	{31., 204.}	106.672	0.329564	newly 54 weeks cumul	236.	215.96	{38., 409.}	51.4831	0.345197
50	newly 3 weeks spec	20.	17.85	{2., 34.}	49.75	0.390445	newly 3 weeks cumul	56.	59.27	{10., 103.}	49.0536	0.296646	newly 5 weeks spec	23.	13.04	{0., 26.}	54.9565	0.580293	newly 5 weeks cumul	95.	87.53	{11., 151.}	46.6421	0.332189	newly 51 weeks cumul	220.	165.31	{15., 328.}	51.7227	0.443511
